# A wearable belt for non-invasive monitoring of heart failure parameters

**DOI:** 10.1016/j.sbsr.2025.100804

**Published:** 2025-05-22

**Authors:** Sheikh M.A. Iqbal, Mary Ann Leavitt, Imadeldin Mahgoub, Waseem Asghar

**Affiliations:** a Department of Electrical Engineering & Computer Science, Florida Atlantic University, Boca Raton, FL 33431, United States of America; b Asghar-Lab, Micro and Nanotechnology in Medicine, College of Engineering and Computer Science, Boca Raton, FL 33431, USA; c Christine E. Lynn College of Nursing, Florida Atlantic University, Boca Raton, FL 33431, United States of America; d Department of Biological Sciences (Courtesy appointment), Florida Atlantic University, Boca Raton, FL 33431, USA

**Keywords:** Heart failure, Thoracic impedance, Heart rate, SPO2, Activity status and ICD

## Abstract

Heart failure is a fatal cardiovascular disease in which the heart is unable to pump sufficient blood to meet the requirements of the body. Traditionally, implantable cardioverter defibrillator (ICD) is used for the monitoring and management of heart failure. However, ICD is an implantable solution that requires surgery and is expensive as well. We have previously developed a wearable device that monitors parameters similar to ICD for the monitoring and management of heart failure. These parameters include heart rate, thoracic impedance and activity status. This paper, will discuss the experimentation and results of the device on heart failure patients. We have used the device to measure heart rate, thoracic impedance, and activity status on heart failure patients with ICDs. The results obtained from our non-invasive device are then compared with those obtained from the ICDs to validate our device. Our non-invasive device has shown to perform close to the ICDs rendering it a cost-effective alternative for heart failure patients.

## 1. Introduction

Heart failure (HF) is a cardiovascular disease in which the heart cannot pump sufficient blood to meet the requirements of the body [1–5]. There are around 60 million people around the world with HF disease and according to a study around 6.7 million Americans had HF in 2020, which has increased from 6 million in 2018 [6,7]. A healthy heart pumps around 55–60 % of the total blood volume inside the heart however an HF-affected heart pumps less blood than this normal threshold [8]. Based on the amount of blood pumped, HF can be categorized into two broader categories: Systolic heart failure and Diastolic heart failure [8,9]. Systolic heart failure is also known as heart failure with reduced ejection fraction (HFrEF) and is a critical stage of HF [8]. In this condition, the heart pumps less than 40 % of the total blood volume [8]. Whereas in diastolic heart failure, also known as heart failure with preserved ejection fraction (HFpEF), heart pumps greater than or equal to 50 % [8]. Traditionally, an implantable cardioverter defibrillator (ICD) is used for the monitoring and management of HF. However, ICD is an implantable solution and hence is expensive. In US, a simple ICD costs on average $18,000 [10]. Moreover, it is only for serious HF patients i.e. HFrEF. An ICD measures different parameters for the monitoring of HF.

These parameters relate to different symptoms of the HF. Some of the important parameters include thoracic impedance (TI), heart rate (HR) and activity status (AS) [1,11–13]. TI is the impedance of the thorax region. On the onset of HF, TI decreases due to the fluid accumulation in the thorax region, whereas the fluid accumulation, usually known as pulmonary edema, is because of the lack of cardiac output from the heart [14]. Similarly, to compensate for the blood supply, a heart failure-affected heart beats at a faster rate than normal (60–120 bpms) whereas due to the lack of blood supply a heart failure patient experiences fatigue and dizziness that reduces the activity status of the subject [15–17]. ICD monitors these significant parameters along with others to monitor and manage heart failure. HF can be better managed with continuous monitoring [18–20]. Continuous monitoring helps in reducing rehospitalization [19]. According to a recent study, hospitalization burden due to HF is very high [21]. Cost per hospitalization due to HF is on average $10,737–$17,830 [21]. As discussed, ICD already an expensive solution, is an implantable device that manages heart failure by monitoring its vital parameters. Wearable devices are non-invasive healthcare devices that offer potential solutions for continuous and real-time monitoring [22–26]. Moreover, wearable devices have the potential for offering telemedicine where they can be used to monitor vital parameters and remotely share them with physicians [27,28].

Vital Patch by VitalConnect is a recent wearable device for remote monitoring. However, it does not measure thoracic impedance which is considered as one of the most significant parameters to HF [29]. About 90 % of hospitalization is due to fluid overload hence monitoring thoracic impedance along with other parameters is critical for HF management [30]. There are some other ongoing developments in this area, including HF management device by Zoll, however this device requires trained staff to monitor the parameters in order to provide alerts to patients [31,32]. We have developed a wearable device that monitor these parameters non-invasively and have been previously tested on healthy subjects in real-time conditions [33]. In this paper, we have performed a preliminary human study and have measured the parameters using our device on HF patients. We have validated the device parameters with the parameters from the ICDs, implanted in these patients. The subsequent paragraphers will discuss the methodology and results for this comparison along with the issues faced during this trial with some future directions.

## 2. Methods

The wearable device previously developed, measures parameters that are significant to heart failure [33]. These parameters include heart rate (HR), activity status (AS), and transthoracic impedance (TTI). These are similar parameters as measured by the ICD. This section will briefly discuss the development of the device and the experimentation protocol that is conducted for the validation of the wearable device with the standard ICD.

### 2.1. Device development

**Fig. 1.**
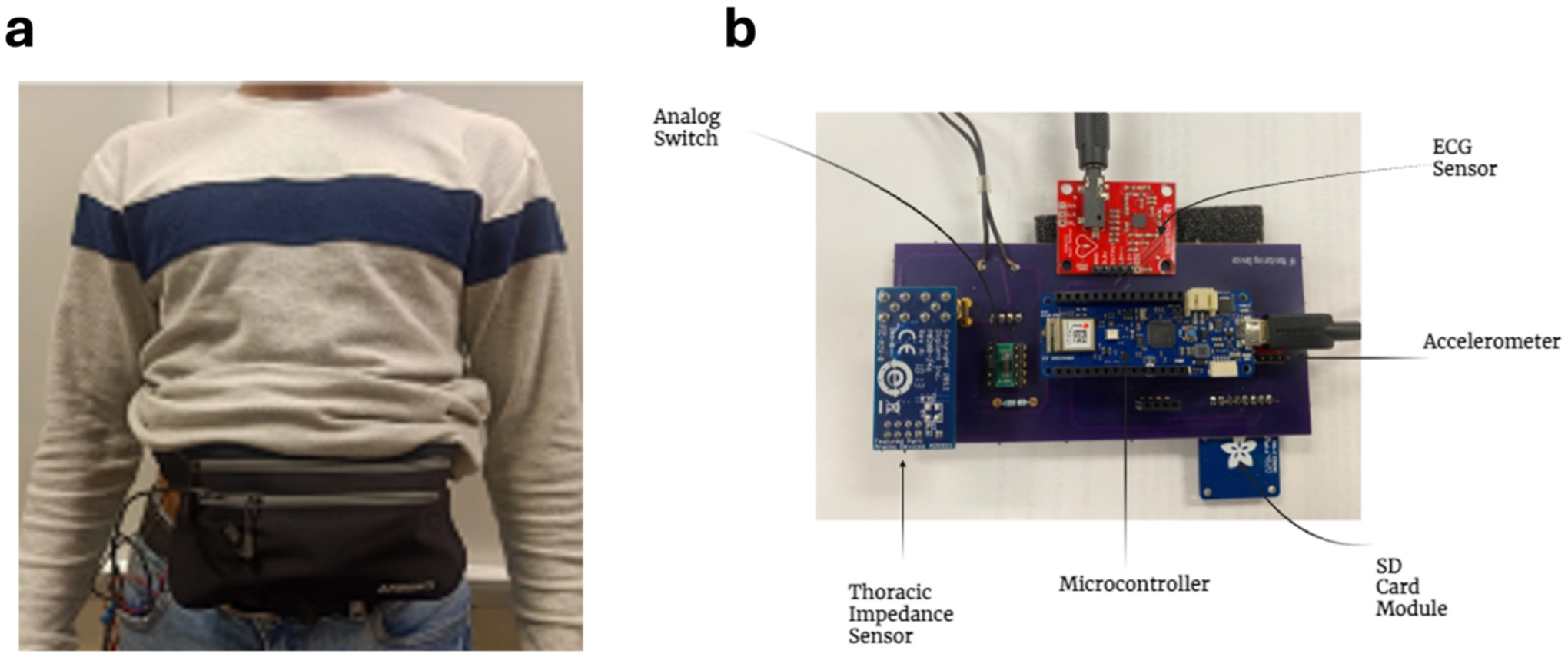
Wearable device for monitoring HF parameters b. Wearable device with different sensors for monitoring HF parameters.

The wearable device can be worn on the abdomen for the detection of parameters such as TI, HR and AS non-invasively (Fig. 1) [33]. As discussed, these parameters are significant for the monitoring and management of HF as they give information regarding different symptoms of HF. For this purpose, we have used different sensors and have embedded them with a microcontroller. These sensors include PMOD Impendence Analyzer (IA) based on AD5933 Integrated Circuit (IC) for the measurement of TI, ADXL 362, an accelerometer, for the detection of activity status and MAX 30105 sensor for the detection of HR [34–37]. AD5933 is a programmable IC that can be used to measure unknown impedances, in our case TI, at set frequency. We have programmed the IC to measure TI from 80 to 100 KHz frequencies, as at these frequencies, TI response is most significant and is considered a safe frequency for injecting currents in human subjects. A two-electrode system is used for the measurement of TI. In this configuration, an AC current is injected into the thorax region from one electrode and the impedance of the region is measured from the other electrode. Moreover, the accelerometer is programmed with timing and activity thresholds to differentiate between active and non-active states of the subject. The MAX 30105 is an optical sensor based on photoplethysmography (PPG) technology. In MAX 30105, an infrared ray (IR) is emitted, and HR is measured based on the rate of absorption of the IR ray by the oxygenated blood particles [38,39].

These sensors are embedded with the Arduino MKR 1010 sensor that allows both I2C and SPI modes of communication for the sensors [40]. The device is then packaged inside a wearable belt that can be conveniently worn on the waist. The device has been validated previously on healthy subjects where expected results for the parameters were obtained [33,41,42].

### 2.2. Experimentation

The device has been used to measure aforementioned parameters in real time and continuously for heart failure patients. These HF patients have reduced ejection fraction with ICDs implanted in them. These patients are enrolled with a cardiologist in a local hospital and implanted ICDs report the parameters to the Medtronic Carelink Portal. To validate the developed device, we have compared the parameters from our wearable device with the parameters measured by the standard ICD in patients. All experiments were conducted with the approval of the Institutional Review Board (IRB) from Florida Atlantic University (FAU). For this purpose, we gave these patients our device for 5 days a week and demonstrated them to use the device for 5 h a day (12–5 pm). As discussed, HF is a progressive disease and therefore for the purpose of validation, we have compared our device with the ICD on multiple days based on their per day average values. Table 1. shows the details of the comparison.

ICD measures intrathoracic impedance (ITI) whereas the wearable device measures the transthoracic impedance (TTI). Both are measures of the thoracic impedance however ITI is an invasive way of measuring thoracic impedance whereas TTI is a non-invasive way of measuring thoracic impedance. The device stores the data in the SD card which was retrieved at the end of the week. The data from the device is then processed to remove any outlier due to noise and is compared with the parameters from the ICD. The comparison of the parameters is done for the same patient at the same day. The subsequent section will discuss the details the comparison.

## 3. Results

**Fig. 2.**
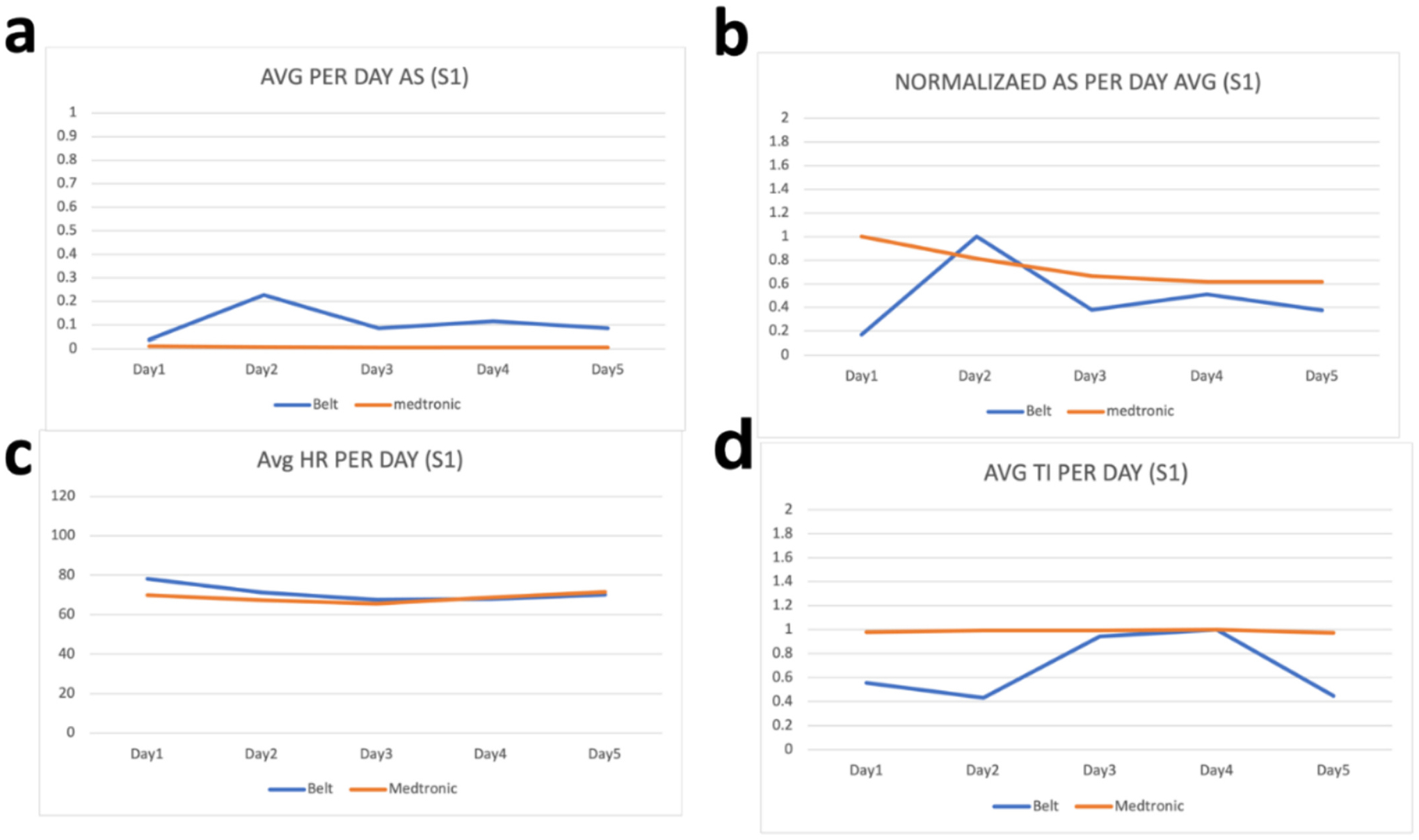
Patient 1 parameters a. Avg AS b. Avg normalized AS c. Avg HR d. Avg TI.

**Fig. 3.**
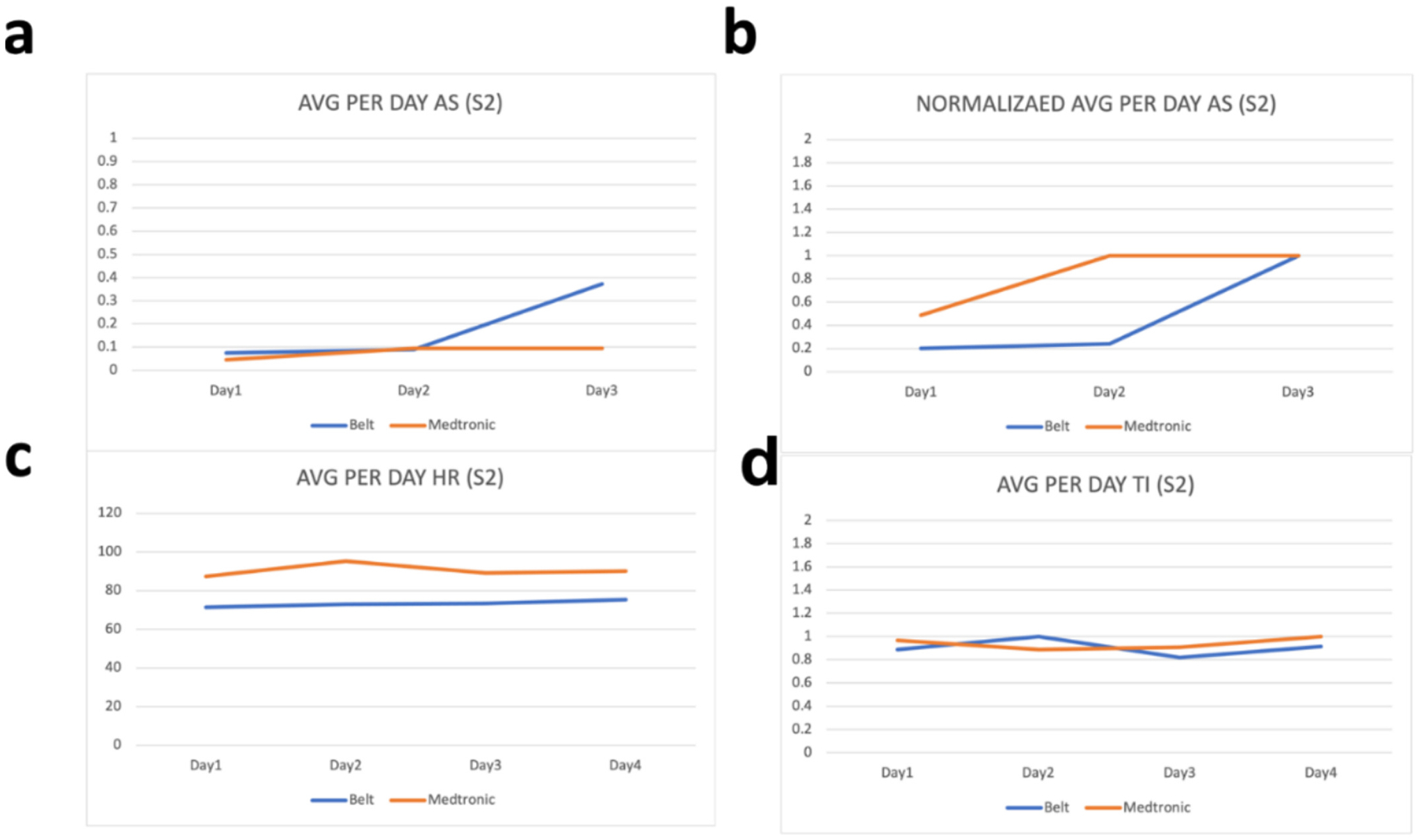
Patient 2 parameters a. Avg AS b. Avg normalized AS c. Avg HR d. Avg TI.

**Fig. 4.**
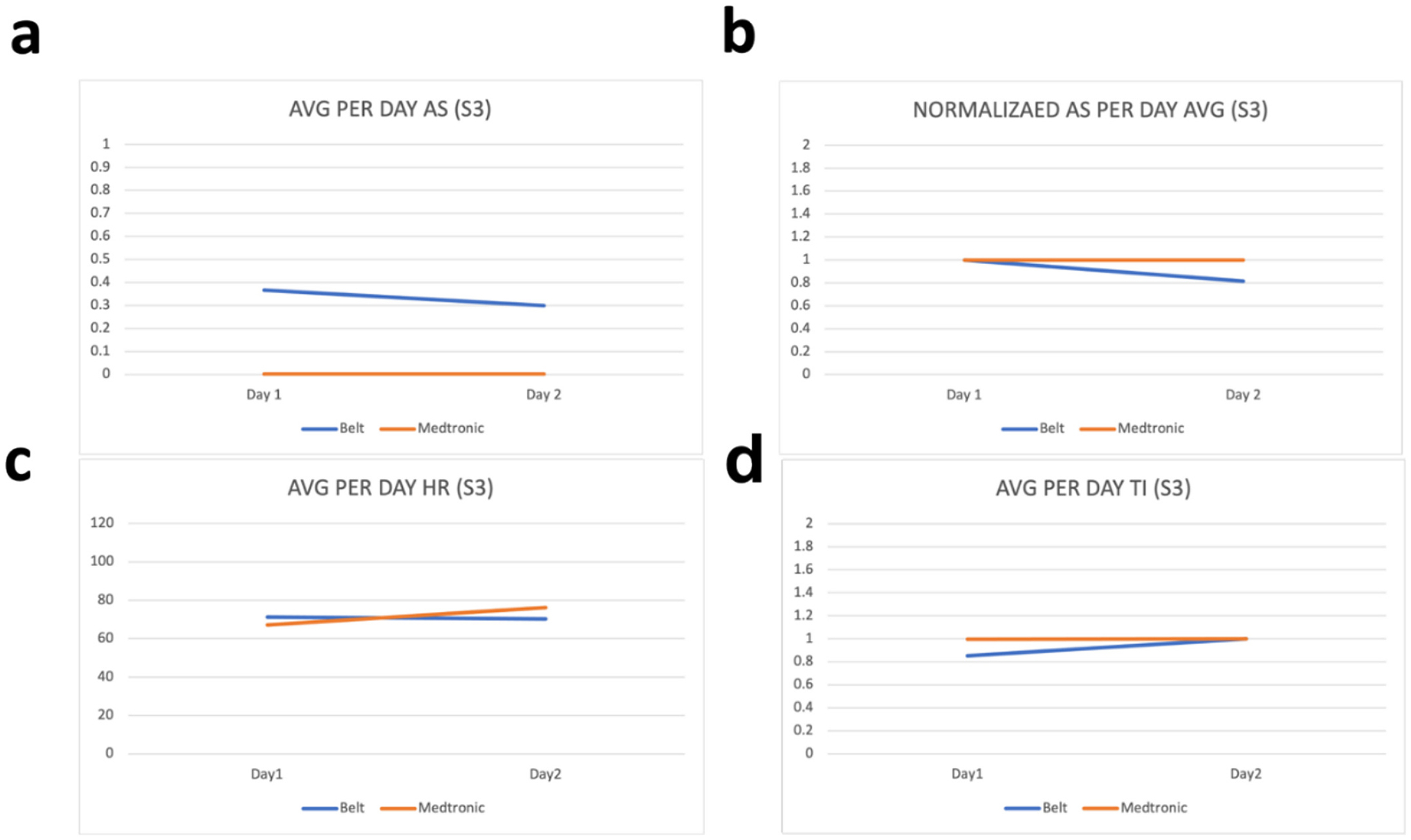
Patient 3 parameters a. Avg AS b. Avg normalized AS c. Avg HR d. Avg TI.

**Fig. 5.**
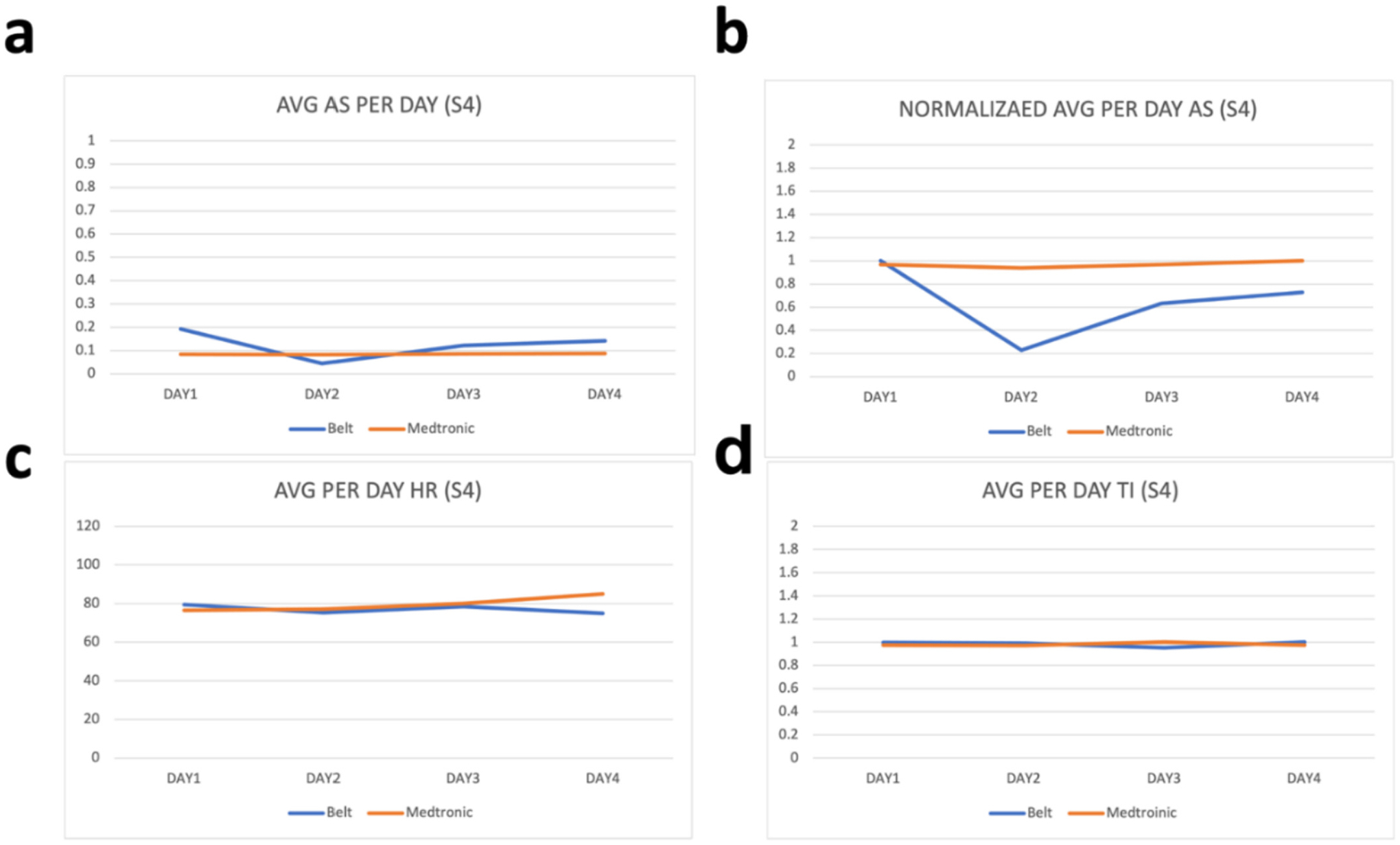
Patient 4 parameters a. Avg AS b. Avg normalized AS c. Avg HR d. Avg TI.

**Fig. 6.**
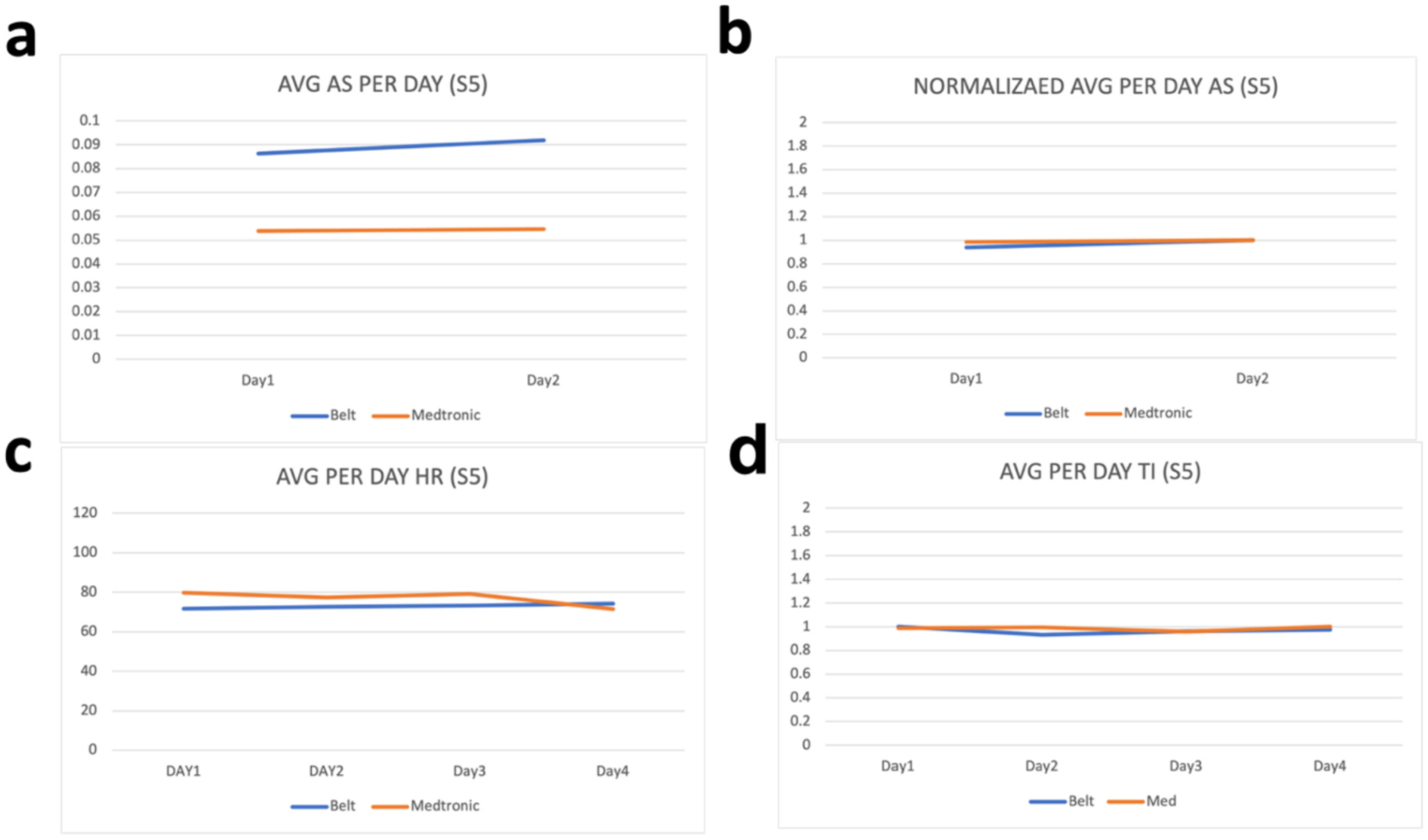
Patient 5 parameters a. Avg AS b. Avg normalized AS c. Avg HR d. Avg TI.

Raw values from the wearable device are processed before their comparison with the parameters from the ICD. This processing includes removal of the noise by filtering values outside the range of values and taking the per day average of the values. These per day averages are then compared with the per day averages of the parameters from the ICD. To evaluate how far or close the parameters of the wearable device are with the parameters of ICD, we have calculated the percentage difference between each parameter. As our device is calibrated in a way that the thoracic impedance exists in between 170 and 800 Ω therefore we have filtered all the values for thoracic impedance that exist outside this range and have then taken per day average of these values. Similarly, as heart rate can vary between 60 and 120 bpm therefore we have filtered all values outside this range and have taken their per day average. Moreover, for activity status, we have coded our sensor to sense activity as 1 and non-activity as 0 and therefore the values shown in Figs. 2–6 for activity status are the average taken per day to reflect how much active the patient has been throughout the day.

We have compared the values obtained by the developed wearable device with respective values on the Medtronic portal for the same patients on the same days. As “AS” on Medtronic portal is given in the number of hours per day, therefore Medtronic AS value is divided by 24 to have the number of times per hour the patient was active during that day. Results can be seen in Fig. 2–6. As thoracic impedance from the belt is transthoracic impedance (TTI) and ICD records intrathoracic impedance (ITI). Both are thoracic impedances (TI) with one measured non-invasively (TTI) and other invasively (ITI). They are on different scales, with TTI containing skin resistance as well. Therefore, we have normalized TTI from our belt and ICD with their respective maximum values during those five days. It can be seen that for some patients, data for full 5 days is not available. This is because of the non-compliance of some patients with the defined experiment. Because experiments were unsupervised and uncontrolled, where patients were briefed on how to use the belt and then were given a belt to use in their home settings therefore some patients were not able to perform the testing on the same day and hence data on these days was either not stored or was not within the acceptable ranges of the parameters. It can be seen from these figures that parameters from the belt are very close to their counterparts on the ICD. Overall, the HR and the TI are more relatable to their respective values on the ICD. The mean percentage differences in the TI and HR values for all patients are less than 10 % except the HR for S3 and TI for S1 whereas the mean percentage difference between AS values is 61.88 %.

The percentage difference for S1 TI is 31.8 % and the percentage difference for S3 HR is 18.95 %. Percentage differences less than or equal to 10 % are considered similar or relatable which shows the similarity in the TI and HR parameters of the belt with the TI and HR parameters from the ICD [43]. To address the high percentage difference for the AS, a further analysis has been performed for the AS. As evident from below graphs the AS from the belt and the Medtronic seems to be on different scales, due to the reason discussed above, therefore we have normalized them to their highest value during the five-day period. It can be seen from Fig. 2–6 d that in comparison to the unnormalized values the normalized AS are closer to each other. This normalization is also helpful in understanding the similarity in the trend of the two activity statues during the 5-day period. Moreover, the percentage difference for the AS has decreased from 61.88 % to 26.37 %. A higher mean percentage difference for AS can also be because of the difference in the definition of the status of activity in the belt and in the ICD. As the values on Medtronic are averages over a day and includes the sleep time as well. During this time AS should be zero and because of this reason AS from belt are generally higher than the AS from ICD. Table 2 shows the details of these patients along with their percentage differences with ICD values.

## 4. Discussion

A wearable belt for the monitoring of heart failure parameters has been demonstrated on actual heart failure patients with reduced Ejection Fraction. These patients have ICDs implanted in them that record similar parameters such as thoracic impedance, heart rate and activity status of the patients and report them on the Medtronic Carelink portal. We have compared our device with patient’s parameters reported by the ICD and have found the parameters from our device to be close to the parameters on ICD. Even though our parameters are taken non-invasively, we have found that our belt reports data trends similar to an invasive ICD. Moreover, it is important to mention here that for HF it is not the absolute values of these parameters that are important but the way they change over a period of time. Therefore, a non-invasive measurement of these parameters with insignificant differences with the parameters from the ICD would be a cost-effective and convenient solution for both the HFrEF and HFpEF HF patients.

In this human study, we have experienced several issues because of the un-supervised and uncontrolled experimentation that has led to the loss of data. A primary issue was the compliance of the patients with the protocol. As most of these patients were elderly therefore despite demonstrating to them personally how to use the device, some of the patients were not able to perform the experiment as guided for all 5 days. Another significant issue was the missing data on the Medtronic portal by the ICD for the same days for patients who have collected data from our device. Moreover, another issue was the detachment of the SD card from the SD card reader which led to the loss of data. It is for these reasons we were not able to get data for all 5 days for all patients.

However, based on the available data we have shown that the values from our device are not significantly different from the values reported by the ICD which shows the potential of the device for the non-invasive measurement of parameters significant towards the HF. The device has the potential to be transformed into a prognostic device from a diagnostic device. For this purpose, the trial on the HF patients’ needs to be extended to have sufficient data for developing a predictive algorithm. The algorithm can then define thresholds for each of the above-mentioned parameters to differentiate the healthy parameters from the HF parameters.

## 5. Conclusion

In this paper, we have validated our wearable device on HF patients. We have used the device to measure heart rate, thoracic impedance, and activity status and compared their values with those measured using an ICD on the HF patients with HFrEF. We have found that our device is able to measure HR with a percentage difference of less than 10 %, TI with a percentage difference of approximately 10 % and a percentage difference of less than 30 % for AS. A percentage difference of less than or near 10 % is not a significant percentage for a smaller dataset to differentiate two parameters and hence demonstrates that the device can measure HR and TI closer to what was reported by the ICD. The developed device after further refinement can potentially be used for measuring HF-related parameters noninvasively.

## Supplementary Material

1

## Figures and Tables

**Table 1:** Comparison of parameters from the ICD and the wearable device.

Patient	Day 1	Day 2	Day 3	Day 4	Day 5	Device
Patient 1	HR_Day_	HR_Day_	HR_Day_	HR_Day_	HR_Day_	ICD
	ITI	ITI	ITI	ITI	ITI	
	AS	AS	AS	AS	AS	
	HR	HR	HR	HR	HR	Wearable device
	TTI	TTI	TTI	TTI	TTI	
	AS	AS	AS	AS	AS	

HR_Day_ = Heart Rate during the day, ITI = Intrathoracic Impedance, TTI = Transthoracic Impedance, AS = Activity Status.

**Table 2:** Percentage differences of AS, HR and TI from belt with their respective parameters from ICD.

Patients	Parameters	Day1	Day2	Day3	Day4	Day5	Mean %age difference	Mean As	Mean AS_Norm	Mean HR	Mean TI
S1	AS	70.57	95.96	91.28	93.98	91.85	80.73	61.88	26.37	8.37	10.89
	HR	10.54	5.70	2.89	1.08	2.02	4.45				
	TI	43.45	56.55	5.07	4.44E-14	54.17	31.85				
	AS	38.06	5.85	5.07			39.43				
	HR	18.22	23.43	17.78	16.39		18.95				
S2	TI	8.41	11.30	10.02	8.73		9.61				
	AS	99.62	99.53				99.57				
	HR	5.75	7.59				6.67				
S3	TI	14.88	0				7.44				
	AS	56.13	46.16	30.43	37.73		42.61				
	HR	3.71	2.14	1.82	11.78		4.87				
S4	TI	2.60	2.04	4.92	2.45		3.00				
	AS	37.63	40.5				39.06				
	HR	10.20	6.25	7.46	3.73		6.91				
S5	TI	1.17	6.52	0.02	2.55		2.57				

## Data Availability

The datasets generated during and/or analyzed during the current study are available from the corresponding author on reasonable request. They are also provided in the supplementary information document.

## Appendix A. Supplementary data

Supplementary data to this article can be found online at https://doi.org/10.1016/j.sbsr.2025.100804.
