# Supplementary material for: A wearable belt for non-invasive monitoring of heart failure parameters
Source: Sens Biosensing Res. Author manuscript; Available in PMC 2026 May 29. (PMC13218682; doi:10.1016/j.sbsr.2025.100804)
Supplement: 1 [file NIHMS2176215-supplement-1.docx]

**A wearable telehealth system for the monitoring of parameters related to heart failure.**

1. **Arduino Code:**

The code used for the wearable device is based on the Arduino and can be accessed here:

https://github.com/ShkhAsher/DEVELOPMENT-OF-A-WEARABLE-DEVICE-FOR-MONITORING-PHYSIOLOGICAL-PARAMETERS-RELATED-TO-HEART-FAILURE
